# Residual phosphorus and zinc influence wheat productivity under rice–wheat cropping system

**DOI:** 10.1186/s40064-016-1907-0

**Published:** 2016-03-01

**Authors:** Amanullah, Inamullah

**Affiliations:** Department of Agronomy, The University of Agriculture, Peshawar, Pakistan; Department of Agriculture Extension, Malakand Batkhela, Khyber Pakhtunkhwa Pakistan

**Keywords:** Grain yield, Yield components, Soil residual P and Zn contents

## Abstract

Continuous cropping of rice (*Oryza sativa* L.) and wheat (*Triticum aestivum* L.) deplete soil fertility and crop productivity. One strategy to increase crop productivity under rice–wheat system is balanced application of crop nutrients. Field experiment was conducted to assess the impact of phosphorus (0, 40, 80, 120 kg P ha^−1^) and zinc (0, 5, 10, 15 kg Zn ha^−1^) on the productivity of rice genotypes (fine and coarse) and their residual effects on the grain yield (GY) and its components (YC) of the succeeding wheat crop under rice–wheat cropping system (RWCS) in North Western Pakistan during 2011–12 and 2012–13. After rice harvest in both years, wheat variety “Siren-2010” was grown on the same layout but no additional P, K and Zn was applied to wheat crop in each year. The GY and YC of wheat significantly increased in the treatments receiving the higher P levels (120 > 80 > 40 > 0 kg P ha^−1^) and Zn (15 > 10 > 5 > 0 kg Zn ha^−1^) in the previous rice crop. The residual soil P and Zn contents after rice harvest, GY and YC of wheat increased significantly under low yielding fine genotype (B-385) as compared to the high yielding coarse genotypes (F-Malakand and Pukhraj). The residual soil P and Zn, GY and of wheat increased significantly in the second year as compared with the first year of experiment. These results confirmed strong carry over effects of both P and Zn applied to the previous rice crop on the subsequent wheat crop under RWCS.

## Background

The rice–wheat cropping system (RWCS) has been in practice in Asia for more than 1000 years. The RWCS covers 13.5 million ha in South Asia: India (10.0), Pakistan (2.2), Bangladesh (0.8) and Nepal (0.5). It represents 32 % of the total rice area and 42 % of the total wheat area in these countries. The productivity of the rice–wheat system remains below the potential yield. The major cause of low yield under RWCS is nutrients depletion from the soil (Dawe 2000; Shah et al. 2011). The continuous RWCS for several decades has thus resulted in nutrients depletion and decline in yields (Zia et al. 1996; Hobbs and Morris 1996; Dawe 2000; Duxbury 2000; Shah et al. 2011).

Phosphorus is second to nitrogen in total application to crops yet is used by plants in much lower quantities. Unlike N, soil P readily forms weakly soluble mineral compounds in the soil, thus resulting in poor mobility and requiring plant roots to explore new regions in the soil to facilitate P uptake (Nichols et al. 2012). Zinc deficiency was first diagnosed in rice on calcareous soils of northern India (Yoshida and Tanaka 1969). It was subsequently found to be a widespread phenomenon in lowland rice areas of Asia, and, next to nitrogen and phosphorus (P) deficiency; Zn deficiency is now considered the most widespread nutrient disorder in lowland rice (Quijano-Guerta et al. 2002).

Phosphorus (P) and zinc (Zn) deficiency are considered the most important nutritional constraints which decrease crop productivity (Ismail et al. 2007; Rose et al. 2013). Most of the researchers confirmed that Zn and P imbalance (Alloway 2009; Khorgamy and Farnis 2009) causes Zn deficiency. The deficiencies of Zn are common in subtropical areas of India, Pakistan, Latin America and Turkey (Cakmak 2008). The deficiency of Zn is considered to loss crops yield up to 40 % (Ozkutlu et al. 2006). The current use of P fertilizer is also inadequate which resulting in P deficiency (Saleque et al. 2006).

Research on P and Zn management under RWCS is lacking. For sustainable rice and wheat production, research on the interactive effects of P and Zn on rice crop and their residual effect on the succeeding wheat crop is needed. This study was therefore conducted to with and objective to find whether P and Zn applied to rice had any significant effects on the productivity of the subsequent wheat crop?

## Methods

### Site description

**Fig. 1:**
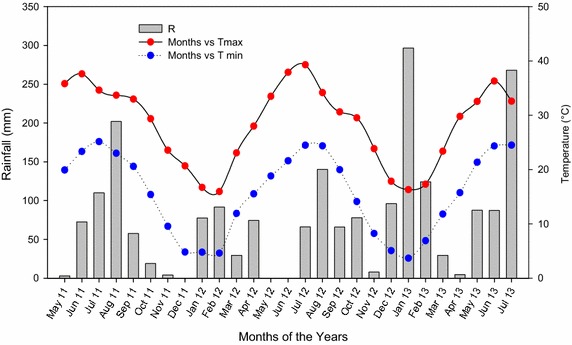
Rainfall and temperature data in the experimental site for the two growing seasons of rice in rice–wheat cropping system

Field experiment was conducted to investigate the impact of zinc (Zn) and phosphorus (P) levels on three rice (*Oryza sativa* L.) genotypes and their residual effects on the GY and YC of subsequent wheat (*Triticum aestivum* L., cv. Siran) under rice–wheat cropping system. The experiment was conducted at Batkhela, Malakand Agency on farmer’s field in Northwest Pakistan during 2011–12 and 2012–13. Batkhela is located at 34°37′0″ N and 71°58′17″ E in degrees minutes seconds (DMS) or 34.6167 and 71.9714 (in decimal degrees). The soil of the experimental site is clay loam, slightly alkaline in reaction (pH = 7.3), non-saline (ECe = 1.02 dS m^−1^), moderately calcareous in nature (CaCO_3_ = 7.18 %) (Gee and Bauder 1986), low in soil fertility containing less organic matter (0.71 %) (Nelson and Sommers 1982), extractable P (5.24 mg kg^−1^) and Zn (0.93 mg kg^−1^) (Soltanpour 1985). Weather data for the rice–wheat cropping system during 2011–12 and 2012–13 is given in Fig. 1.

## Experimentation

### Rice crop

The factorial experimental treatments were four phosphorus (P) levels (P_1_ = 0, P_2_ = 40, P_3_ = 80 and P_4_ = 120 kg P ha^−1^) and three rice genotypes (G_1_ = Bamati-385 (fine), G_2_ = Fakhre-e-Malakand (coarse) and G_3_ = Pukhraj (coarse)) kept in main plots, while four Zn levels (Zn_1_ = 0, Zn_2_ = 5, Zn_3_ = 10 and Zn_4_ = 15 kg Zn ha^−1^) as sub plots. The experiment was carried out in randomized complete block design with split-plot arrangement having three replications. A sub-plot size of 12 m^2^ (3 m × 4 m) consisting of 25 hills m^−2^ with hill to hill distance of 20 cm apart was used. A uniform dose of 120 kg N ha^−1^ as urea [CO(NH_2_)_2_] having 46 % N, and 60 kg K_2_O ha^−1^ in the form of potassium chloride [KCl] were applied uniformly to all plots. All the required K, P and Zn were applied at the time of transplanting, while nitrogen (N) as urea was applied in two equal splits i.e. 50 % each at 0 and 30 days after transplanting. Phosphorus was applied in the form of triple super phosphate [Ca(H_2_PO_4_)_2_] consisted of 46 % P_2_O_5_, and zinc was applied in the form of zinc sulphate [ZnSO_4_H_2_O] consisted of 36 % zinc and 18 % sulphur. The amount of sulfur (S) was maintained constantly in the Zn applied plots by adding additional sulphur using sulphate of potash [K_2_SO_4_] which contains 50 % K_2_O and 18 % sulphur. All subplots were separated by about 30 cm ridges to stop movement water/nutrient among different treatments.

### Soil analysis

After rice harvest in October in both years, soil samples were taken (15 cm depth) from each treatment, AB-DTPA-extractable P and AB-DTPA-extractable Zn concentrations in soil samples were determined according the standard procedures of Soltanpour (1985).

### Wheat crop

Wheat variety Siren-2010 was planted on same layout of the previous rice crop. No additional P, K and Zn were applied to wheat crop. Nitrogen at 140 kg N ha^−1^ in the form of urea was applied in three splits i.e. 1/3rd each at sowing, 30 days after emergence and booting stage.

### Data handling and recording

Data on various yield and yield components parameters (spikes m^−2^, grains spike^−1^, 1000-grains weight, and grain yield) of subsequent wheat crop was recorded. Number of spikes were counted in two rows each two meter long at two different places in each sub-plot, and then converted into number spikes m^−2^. Number of grains was counted in 10 randomly selected spikes in each sub-plot and then averaged grains spike^−1^ was calculated. The four central rows each three meter long in each treatment were harvested at maturity, the materials was dried, threshed; grains were separated, cleaned, weighed and converted into grain yield (kg ha^−1^). Thousand grains from each sub-plot were counted and weighed in grams by using the electronic balance.

### Statistical analysis of data

The factorial experimental treatments for the preceding rice crop were four phosphorus (P) levels (P_1_ = 0, P_2_ = 40, P_3_ = 80 and P_4_ = 120 kg P ha^−1^) and three rice genotypes (G_1_ = Bamati-385 (fine), G_2_ = Fakhre-e-Malakand (coarse) and G_3_ = Pukhraj (coarse)) used as main plots factor, while four Zn levels (Zn_1_ = 0, Zn_2_ = 5, Zn_3_ = 10 and Zn_4_ = 15 kg Zn ha^−1^) were used as sub plot factor. The experiment was carried out in randomized complete block design with split-plot arrangement having three replications. After rice harvest, wheat variety “Siren-2010” was planted on same layout of the previous rice crop. Data on all parameters were subjected to analysis of variance (ANOVA) according to the methods described for randomized complete block design with split plot arrangement combined over the years (Steel et al. 1996), and means between treatments were compared using LSD (least significant difference) test (*p* ≤ 0.05).

## Results

### Soil P and Zn contents after rice harvest

**Fig. 2:**
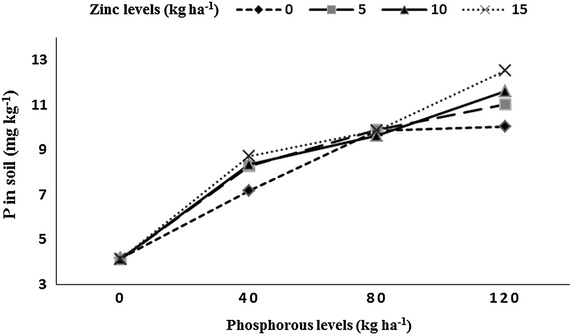
Residual soil phosphorous (mg kg^−1^) after rice harvest as affected by residual soil phosphorus into zinc (P × Zn) interaction

**Fig. 3:**
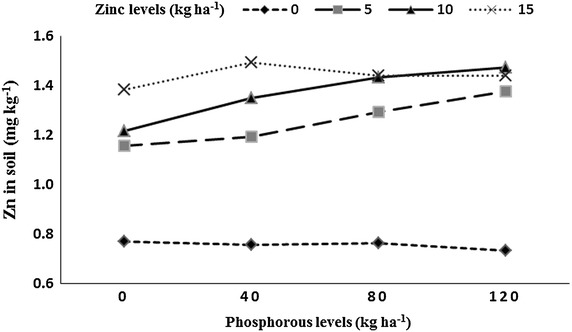
Residual soil zinc (mg kg^−1^) after rice harvest as affected by residual soil phosphorus into zinc (P × Zn) interaction

Residual soil AB-DTPA-extractable P and AB-DTPA-extractable Zn was significantly affected by P and Zn levels, P × Zn, preceding rice genotypes and years (Table 1). The 2 years mean data indicated that highest P concentration (11.3 mg kg^−1^) was observed with 120 kg P ha^−1^, while lowest P concentration (4.1 mg kg^−1^) was observed in the P control plots. In case of Zn levels, the highest P concentration (8.8 mg kg^−1^) was recorded with 15 kg Zn ha^−1^, while lowest P concentration (7.8 mg kg^−1^) was recorded in Zn control plots. Among rice genotypes, P concentration was increased to maximum (9.1 mg kg^−1^) under fine rice genotype (B-385). The lowest P concentration (7.8 mg kg^−1^) was observed under high yielding rice hybrid (Pukhraj). P concentration in soil increased in year two (8.8 mg kg^−1^) than year one (7.9 mg kg^−1^). Increase in both P and Zn levels to rice crop increased soil P content (Fig. 2). The highest soil Zn content (1.26 mg kg^−1^) was noted with 120 kg P ha^−1^ being at par 80 kg P ha^−1^ (1.23 mg kg^−1^), the lowest residual soil Zn concentration (1.13 mg kg^−1^) was observed in plots receiving no P. Soil Zn content increased (1.44 mg kg^−1^) with 15 kg Zn ha^−1^, while minimum Zn soil concentration (0.75 mg kg^−1^) was recorded under Zn control plots. The highest residual soil Zn concentration (1.34 mg kg^−1^) after rice harvest was recorded in pots under B-385, followed by F-Malakand (1.19 mg kg^−1^), while minimum (1.08 mg kg^−1^) was observed under Pukhraj. Soil Zn content was significantly higher (1.24 mg kg^−1^) in year two than year one (1.17 mg kg^−1^). Residual soil Zn increased with application of 15 kg Zn ha^−1^ under P control plots or the plots that received 40 kg P ha^−1^ (Fig. 3).

**Table 1:** Residual soil phosphorous (P) and zinc (Zn), number of spikes m^−2^, number of grains spike^−1^, thousand grains weight and grain yield of wheat as affected by preceding rice genotypes, phosphorus and zinc levels under rice–wheat cropping system

Applied (kg P ha^−1^)	Residual soil P (mg kg^−1^)	Residual soil Zn (mg kg^−1^)	Number of spikes m^−2^	Number of grains spike^−1^	Thousand grains weight (g)	Grain yield (kg ha^−1^)
0	4.1 d	1.13 c	226 d	45 d	36.06 c	3434 d
40	8.1 c	1.20 b	256 c	49 c	39.08 b	3974 c
80	9.8 b	1.23 ab	282 b	51 b	40.46 a	4194 b
120	11.3 a	1.26 a	290 a	52 a	40.54 a	4291 a
LSD_0.05_	0.23	0.030	5.13	0.7	0.578	72.34
Applied Zn (kg ha^−1^)						
0	7.8 c	0.75 d	253 c	48 c	38.51 c	3800 c
5	8.3 b	1.26 c	261 b	49 b	39.25 ab	3965 b
10	8.4 b	1.37 b	268 a	50 a	39.53 a	4010 b
15	8.8 a	1.44 a	271 a	50 a	38.86 bc	4118 a
LSD_0.05_	0.19	0.027	4.55	0.5	0.455	75.70
Rice genotypes						
Basmati-385	9.1 a	1.34 a	277 a	50 a	39.47 a	4234 a
F-Malakand	8.1 b	1.19 b	270 b	50 a	39.19 a	4085 b
Pukhraj	7.8 c	1.08 c	243 c	48 b	38.44 b	3601 c
LSD_0.05_	0.20	0.026	4.44	0.6	0.500	62.65
Years						
Year-I	7.9 b	1.17 b	261 a	49 b	38.73 b	3850 b
Year-II	8.8 a	1.24 a	265 a	50 a	39.34 a	4097 a
P × Zn	**Fig 2	**Fig 3	**Fig 4	**Fig 5	*Fig 6	***Fig. 7

Means of the same category followed by different letters are significantly different at 5 % level of probability using LSD test

ns stands for non-significant, while *, ** and *** stands for significant at 5, 1 and 0.1 % level of probability, respectively

### Yield and yield components of wheat

**Fig. 4:**
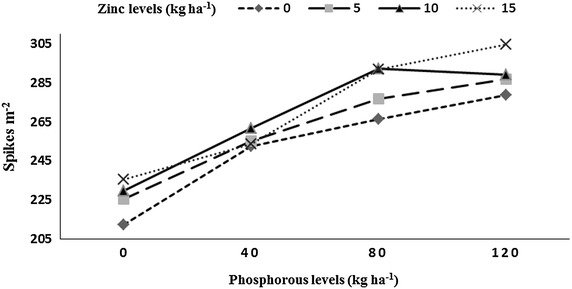
Number of spikes m^−2^ of wheat as affected by residual soil phosphorus into zinc (P × Zn) interaction

**Fig. 5:**
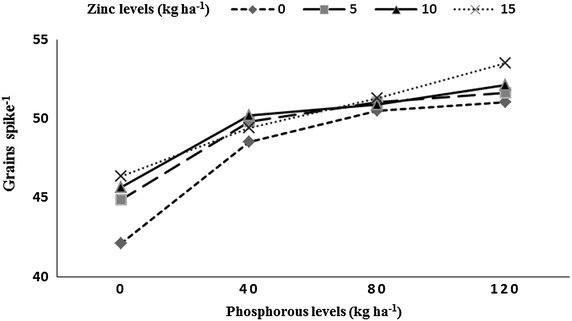
Number of grains spike^−1^ of wheat as affected by residual soil phosphorus into zinc (P × Zn) interaction

Number of spikes m^−2^, grains spike^−1^, thousand grains weight and grain yield was significantly affected by residual P and Zn levels, P × Zn, and preceding rice genotypes (Table 1). Highest number of spikes m^−2^ (290) were produced by wheat with higher residual soil P (under 20 kg P ha^−1^ was applied to rice), while minimum spikes m^−2^ (226) were recorded with low residual soil under P control plots for the previous rice crop. Highest spikes m^−2^ (271) were recorded with highest residual soil Zn (15 kg Zn ha^−1^ applied to rice), while minimum spikes m^−2^ (253) were recorded under the plots where the previous rice crop received no Zn (control). Wheat grown after fine genotype (B-385) had more number of spikes m^−2^ (277), while significant reduction in spikes m^−2^ (243) was observed under hybrid rice (Pukhraj). The P × Zn interaction indicated that increase in residual soil P and Zn increased spikes m^−2^ in wheat (Fig. 4). Increase in the residual soil P increased number of grains spike^−1^ (52) in wheat, while minimum grains spike^−1^ (45) were recorded with low residual soil P. Highest number of grains spike^−1^ (50) were produced with the highest residual soil Zn, while minimum grains spike^−1^ (48) were recorded with low residual soil Zn. Wheat grown after B-385 and F-Malakand had higher number of grains spike^−1^ (50 each), while wheat grown after Pukhraj had significantly less number of grains spike^−1^ (48). In year two wheat had more number of grains spike^−1^ (50) than year one (49). Interaction between P × Zn indicated that increase in the residual content of both nutrients (P and Zn) increased grains spike^−1^ of subsequent wheat crop (Fig. 5) and vice versa.

**Fig. 6:**
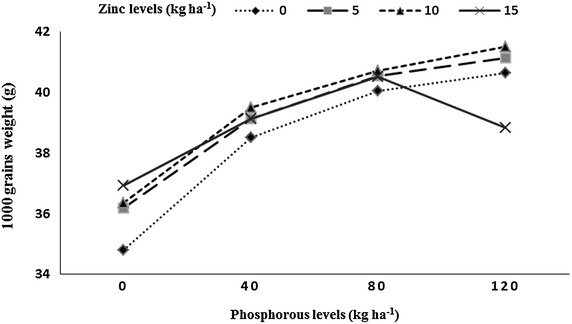
Thousand grains weight (g) of wheat as affected by residual soil phosphorus into zinc (P × Zn) interaction

**Fig. 7:**
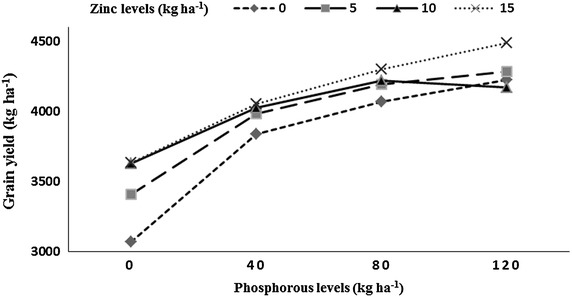
Grain yield (kg ha^−1^) of wheat as affected by residual soil phosphorus into zinc (P × Zn) interaction

Heavier grains (40.54 g/1000 grains) for wheat were recorded when grown under the plots that received the highest P (120 kg ha^−1^) to the previous rice. Minimum thousand grains weight (36.06 g) of wheat crop was recorded in plots where P was not applied to the preceding rice crop. The highest thousand grains weight (39.53 g) was produced by wheat grown under 10 kg Zn ha^−1^ and 15 kg Zn ha^−1^ applied to rice crop, while minimum thousand grains weight (38.51 g) was recorded in Zn control plots. Wheat grown after B-385 had higher thousand grains weight (39.47 g) being at par with wheat grown after F-Malakand (39.15 g). Wheat grown after rice Pukhraj had less thousand grains weight (38.44 g). In year two wheat crop had heavier thousand grains (39.34 g) than in year one (38.73 g). Interaction between P × Zn indicated that at lower Zn levels (0, 5 and 10 kg Zn ha^−1^) thousand grains weight of subsequent wheat crop increased while increasing P level (Fig. 6); when the highest Zn level (15 kg Zn ha^−1^) was applied then increase in P up to 80 kg ha^−1^ increased thousand grains weight and further increase in P level decreased the grains weight. Highest grain yield (4291 kg ha^−1^) for wheat was recorded when grown on the plots where the previous rice crop received the highest of 120 kg P ha^−1^, while minimum grain yield (3434 kg ha^−1^) was recorded in P control plots. Maximum grain yield (4118 kg ha^−1^) was produced by wheat under 15 kg Zn ha^−1^ applied to the previous rice crop, while minimum grain yield (3800 kg ha^−1^) was recorded in Zn control plots. Wheat grown after the low yielding rice genotype B-385 had higher grain yield (4234 kg ha^−1^), followed by wheat grown after F-Malakand (4085 kg ha^−1^), while wheat grown after hybrid rice (Pukhraj) reduced grain yield (3601 kg ha^−1^). Year two had resulted in more grain yield (4097 kg ha^−1^) in subsequent wheat than in year one (3850 kg ha^−1^). Interaction between P × Zn indicated that increase in levels of both nutrient P and Zn for the previous rice crop resulted in higher grain yield in the subsequent wheat crop (Fig. 7).

## Discussion

The increase in P and Zn levels to the preceding rice crop increased the residual soil P and Zn contents that had positive impact on the yield and yield components of the subsequent wheat crop. This indicates the strong carry over effect of both P and Zn for the subsequent wheat crop under rice–wheat cropping system. Likewise, Lal et al. (2000) found that available P in soil samples after harvest increased considerably with increasing rates of P and Zn application. Rehman et al. (2007) reported the highest value (10.95 mg P kg^−1^) of residual soil P with application of higher P level to sorghum. Elsokkary et al. (1981) observed an increase in extractable soil P with the higher P rate. They described that the extent of increase in the available soil P was greater with the application of Zn than Zn-control plots (Zn not applied). According to Elsokkary et al. (1981), application of higher P level to soil significantly increased the availability of Zn to crops. However, the degree of the increase was higher in the soils where Zn was not applied than Zn treated soils.

Highest yield and yield components for the subsequent wheat crop under more residual soil P (11.3 and 9.8 mg kg^−1^) was due to the higher P levels (120 and 80 kg P ha^−1^, respectively) applied to the preceding rice crop. According to Cooke (1982), the recovery of applied phosphatic fertilizer by plants is very low as compared with other nutrients, and only 10–20 % of applied P-fertilizer is available to the current crop, and the residual P is available to subsequent crops (Wild 1988). Phosphate fertilizers can have long lasting residual effects on succeeding crops and due to accumulated residues, the level of soil P gradually raises contributing more to P pool available to growing plants (Harapiak and Beaton 1986). According to Karamanos et al. (2007), P recovery by crops in the year of fertilizer application was very low (10 to 30 %) which depends on soil, crop and management factors. They reported negligible benefits to the residual soil P, so continuous P fertilize was required to increase crop yield.

The highest yield and yield components in subsequent wheat crop was due to the higher residual soil Zn (1.44 and 1.37 mg kg^−1^) extracted from the plots where higher Zn levels (15 and 10 kg Zn ha^−1^, respectively) was applied to the preceding rice. According to Rashid (2005), application of soil applied micronutrients leave a useful residual effect on successive crops. This was because the first crop removes only a small fraction of the applied micronutrient dose. The clayey, alkaline and calcareous nature of soils in Pakistan, Zn fertilizer is mainly fixed by soil particles and very low amount is available and uptake by rice crop plants (Tahir et al. 1991) and so the residual Zn in soils could increase productivity of subsequent wheat crop under rice–wheat cropping system. Zia et al. (1996) reported that residual effect of the higher rate of 10 kg Zn ha^−1^ was most beneficial to increase rice GY than the low rate of 5 kg Zn ha^−1^ under RWCS. Likewise, Hussain (2004) reported that the residual Zn (5 kg ha^−1^) increased the paddy GY by 6.1 %. According to Khan et al. (2009), grain yield, 1000 grains weight and spikes m^−2^ of wheat obtained due to residual application of 10 kg Zn ha^−1^ was higher than residual effect of 5 kg Zn ha^−1^. This indicated that the higher level of Zn applied to the previous crops increase the residual soil Zn for the succeeding crops and hence crop productivity increased. The high residual Zn concentration in soil remained from previous crop increased GY of the succeeding crop on soils having Zn deficiency (Graham and Rengel 1993).

The residual soil P (9.1, 8.1 and 7.8 mg P kg^−1^) and Zn contents (1.34, 1.19 and 1.08 mg Zn kg^−1^) were extracted after the harvesting of previous rice genotypes B-385 (fine), F-Malakand (coarse) and Pukhraj (coarse), respectively. These results confirmed that the two coarse rice genotypes took more P and Zn from the soil (data not shown) and therefore reduced the residual soil P and Zn contents in the soil that had negative impact on the succeeding wheat crop. On the other hand, the fine genotype took less amount of P and Zn from the soil (data not shown) and thereby increased the residual soil P and Zn for the subsequent wheat crop that had positive impact on wheat productivity. Yield and yield components of the subsequent wheat crop were significantly more with higher residual soil P and Zn contents under fine rice genotype in rotation than coarse rice genotypes. Recently, Hussain et al. (2012) found that wheat yield after Super Basmati ranged from 3.08 to 3.98 t ha^−1^ with an average of 3.4 t ha^−1^ while after rice-386 it ranged from 2.2 to 4.5 t ha^−1^ with an average of 3.78 t ha^−1^. Yield and yield components of subsequent wheat were significantly more in year two than in year one. The difference in the yield and yield components of wheat in both years might be attributed to the fluctuation in rainfall data as well as change in soil fertility status in the 2 years. The higher yield and yield components in year two was due to the higher residual soil P and Zn than in year one.

## Conclusions

Nutrient management is very important for improving crop productivity in cereal based system. Our results confirmed that wheat grown on plots having more residual soil P and Zn had positive impact on the GY and YC of subsequent wheat under RWCS. The residual soil P and Zn was increased with increase in P and Zn to the previous rice crop. The high yielding coarse rice genotypes (F-Malakand and Pukhraj) took more P and Zn from the soil and decreased their residual soil contents Subsequent wheat crop productivity decreased tremendously when grown after the high yielding coarse rice genotypes than grown after the low yielding fine rice genotype. Therefore, it was suggested that wheat grown after high yielding genotype either needs an additional P and Zn application or higher rates of both P and Zn could be applied to the preceding rice crop for increasing wheat productivity under RWCS.
